# Causes and factors related to dopamine agonist withdrawal in Parkinson′s disease

**DOI:** 10.1002/brb3.453

**Published:** 2016-05-05

**Authors:** Ester Suárez Castro, Diego Santos‐García, Teresa de Deus Fonticoba, Irene Expósito Ruíz, Cintia Tuñas Gesto, Mercedes Macías Arribí

**Affiliations:** ^1^ Section of NeurologyComplejo Hospitalario Universitario de Ferrol (CHUF)Hospital A. MarcideFerrolSpain

**Keywords:** Age, comorbidity, dopamine agonist, Parkinson′s disease, polypharmacy, tolerability

## Abstract

**Background:**

Although dopamine agonists (DAs) are useful in Parkinson′s disease (PD), they are not frequently used in elderly patients due to adverse effects. However, there is a lack of evidence because few elderly PD patients are enrolled in clinical trials.

**Aims of the study:**

The aims of this study were to analyze the reasons of DA withdrawal (DAW) in a group of PD patients in clinical practice and to identify the related factors. Specifically, we studied the effect of age, comorbidity, and polypharmacy as potential risk factors for DAW.

**Methods:**

A retrospective chart review of the follow‐up (from May, 2012 to March, 2015) of a subgroup of PD patients receiving a DA (*n* = 68; 60.3% males, 69.3 ± 9.2 years old) from a cohort (*n* = 150) previously studied in detail in 2012 was used to identify predictive factors of DAW.

**Results:**

The DAW percentage was 18.2% (12/66; follow‐up of 690.2 ± 232.6 days). DAW causes were cognitive impairment (3), reduction therapy (3), hallucinations (2), dyskinesia (2), and excessive diurnal somnolence (2). Only a higher levodopa daily dose (HR 1.003; 95% CI 1.001–1.006; *P* = 0.044) was an independent predictor of DAW after adjustment for other explanatory variables.

**Conclusions:**

The frequency of DAW was low. Advanced age alone is not a contraindication to the administration of DAs.

## Introduction

Levodopa is the gold standard symptomatic treatment in Parkinson′s disease (PD), although its long‐term use is associated with the development of motor complications. Alternatively, dopamine agonists (DAs) have shown to be effective, both as monotherapy in the early stages of PD or in association with levodopa in advanced PD (Antonini et al. 2009). However, DAs are associated with a greater risk of developing dopaminergic side effects (somnolence, hallucinations, edema, impulse control disorders, etc.) compared with levodopa, and their use is avoided in the elderly for reasons including altered drug metabolism, an increased risk of adverse effects, increased comorbid conditions, increased risk of drug interactions, and a higher risk of cognitive problems and behavior disorders in PD patients over the age of 70 years (Kempster et al. 2007; Stowe et al. 2008). However, there is no compelling evidence for such concerns because few elderly PD patients have been enrolled in clinical trials (Mitchell et al. 1997). Moreover, a previous study demonstrated that trials with DAs are warranted in selected very elderly patients (Shulman et al. 2000).

The aims of this study were as follows: (1) to determine the reasons of DA withdrawal (DAW) in a group of PD patients in clinical practice; and (2) to identify the predictive factors of DAW. Specifically, we studied the effect of age, comorbidity, and polypharmacy as potential risk factors for DAW.

## Materials and Methods

**Figure 1:**
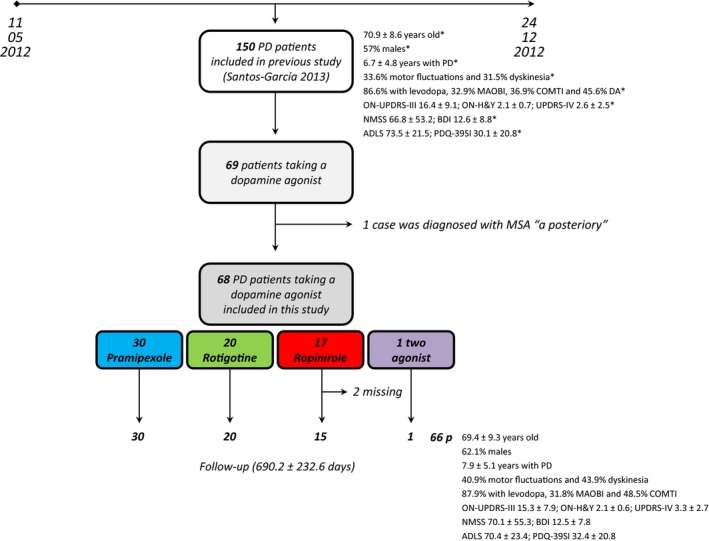
Flowchart of inclusion in the clinical practice cohort. ADLS, Activities of Daily Living Score; BDI, Beck Depression Inventory; COMTI, catechol‐O‐methyl transferase inhibitor; DA, dopamine agonist; DAW, dopamine agonist withdrawal; MAOBI, monoamine oxidase B inhibitor; MSA, multiple system atrophy; NMSS, Nonmotor Symptoms Scale; UPDRS, Unified Parkinson's Disease Rating Scale (part III, motor examination; part IV, motor complications). *Data are from 149 patients because one case was diagnosed with MSA “a posteriori”.

We conducted a retrospective chart review of the follow‐up of a subgroup of nondemented PD patients receiving a DA (*n* = 68; 60.3% males, 69.3 ± 9.2 years old) from a cohort (*n* = 150) previously studied in detail (Santos‐García and de la Fuente‐Fernández 2013, 2015). The patients from the cohort who were receiving a DA, such as pramipexole, ropinirole, and rotigotine at baseline (cross‐sectional study conducted in 2012) were included in this study. Figure 1 shows a flowchart of the selection and inclusion of patients. Baseline assessment included motor dysfunction (ON‐state Hoehn & Yahr [H&Y] / Unified Parkinson's Disease Rating Scale [UPDRS] part III and motor complications [UPDRS part IV]), mood (Beck Depression Inventory [BDI]), nonmotor symptoms (Nonmotor Symptoms Scale [NMSS]), disability (Schwab & England Activities of Daily Living Scale [ADLS]), socio‐demographic variables, and other disease‐related variables.

The follow‐up information was collected from medical records by a neurology resident (E.S.C.). The follow‐up period was from the baseline evaluation (between 11 May and 24 December 2012 depending on the case) to 23 March 2015. The reason for DAW, age at DAW, follow‐up time, time until DAW (time from baseline to DA withdrawal), time taking DA therapy (time taking the DA before baseline) and DA dose (Tomlinson et al. 2010) changes were collected. The study (CADAW‐PD, Causes and fActors related to Dopamine Agonist Withdrawal in Parkinson′s Disease; DIE‐LEV‐2015‐01) was approved by the local ethics committee. All participants signed an informed consent form.

Data were processed using SPSS 21.0 for Windows. Proportions between groups were compared using the chi‐square test. Continuous variables are expressed as the mean ± SD or median and quartiles, depending on whether they were normally distributed and were compared using the Student′s *t* test or the Mann–Whitney test, as appropriate. We defined two groups relative to the primary endpoint (DA withdrawal): (1) patients who discontinued DA therapy during follow‐up (DAW group); and (2) patients who did not discontinue DA therapy (non‐DAW group). Univariate and multiple regression analyses were performed to evaluate predictors of DAW. Specifically, we studied the effect of age, comorbidity, and polypharmacy as potential risk factors for DAW. The number of drugs different of antiparkinsonian agents taken at baseline was considered a surrogate marker of comorbidity. Polypharmacy was assessed by counting the total number of pills at baseline. Values of p < 0.05 were considered significant.

## Results

**Figure 2:**
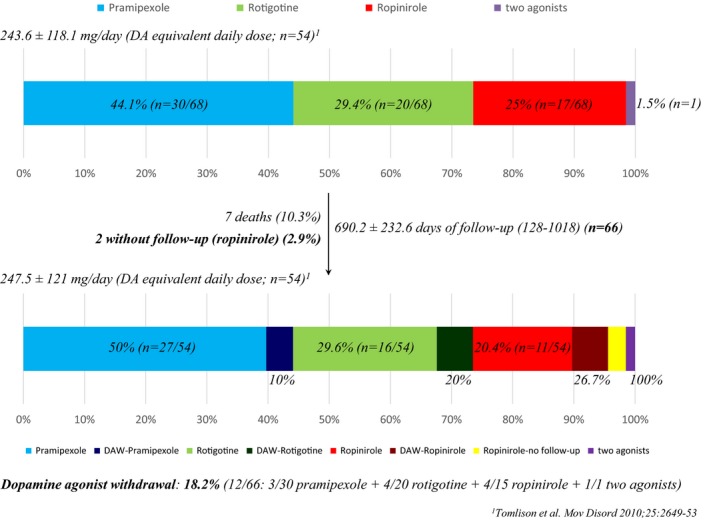
Percentage of patients taking DA therapy (pramipexole, rotigotine and/or ropinirole) at baseline (top bar) and at the end of follow‐up (bottom bar). Cases who discontinued DA therapy are indicated by a dark color in the bottom bar. The DAW percentage was 10% for pramipexole (3/30), 20% for rotigotine (4/20), 26.7% for ropinirole (4/15) and for the only case taking pramipexole + rotigotine. At the end of the follow‐up, 54 patients were receiving DA therapy: 50% pramipexole (27/54), 29.6% rotigotine (16/54) and 20.4% ropinirole (11/54). DA, dopamine agonist.

Sixty‐eight PD patients from the cohort (*n* = 150) (Santos‐García and de la Fuente‐Fernández 2013, 2015) were receiving DA therapy at baseline: 30 pramipexole, 20 rotigotine, 17 ropinirole, and one pramipexole + rotigotine. Seven patients died and two cases (taking ropinirole) were lost to follow‐up. Twelve of 66 patients (18.2%) discontinued DA therapy during follow‐up (690.2 ± 232.6 days; range, 128–1080). Figure 2 shows the frequency of DAW according to DA therapy and the patients' medications at baseline. The reasons for DAW are shown in Table 1.

**Table 1:** Reasons of DAW considering different type of DA therapy. “Time until DAW” refers to time from baseline to DA withdrawal. “Time taking DA therapy” refers to time taking the DA before baseline

DA Therapy	Reasons of DAW	Time until DAW (days)	Age at DAW	Time taking DA therapy (days)
Pramipexole (*n* = 30)	3 cases (10%)	437.7 ± 476.2	71.3 ± 7	1250.3 ± 724.8
	Case 1 simplification of treatment (DBS)	12	64	1987
	Case 2 diurnal somnolence	349	78	1226
	Case 3 starting with levodopa enteral	882	72	538
Rotigotine (*n* = 20)	4 cases (20%)	511.7 ± 333.9	68.8 ± 6.3	548.2 ± 563.3
	Case 1 cognitive impairment ‐> dementia	112	66	48
	Case 2 skin reaction and diurnal somnolence	644	67	155
	Case 3 cognitive impairment ‐> dementia	719	78	723
	Case 4 visual hallucinations	846	64	1267
Ropinirole (*n* = 15)	4 cases (26.7%)	503 ± 118.2	60.3 ± 11.7	1295.5 ± 1052.2
	Case 1 visual hallucinations	385	62	1232
	Case 2 dyskinesia	429	63	2689
	Case 3 simplification of treatment	554	72	1126
	Case 4 dyskinesia/dystonia (ON)	644	44	135
Pramipexol + rotigotine (*n* = 1)	1 case (100%)	551	70	1815
	Cognitive impairment ‐> dementia			

DA, dopamine agonist; DBS, deep brain stimulation; DAW, dopamine agonist withdrawal.

**Figure 3:**
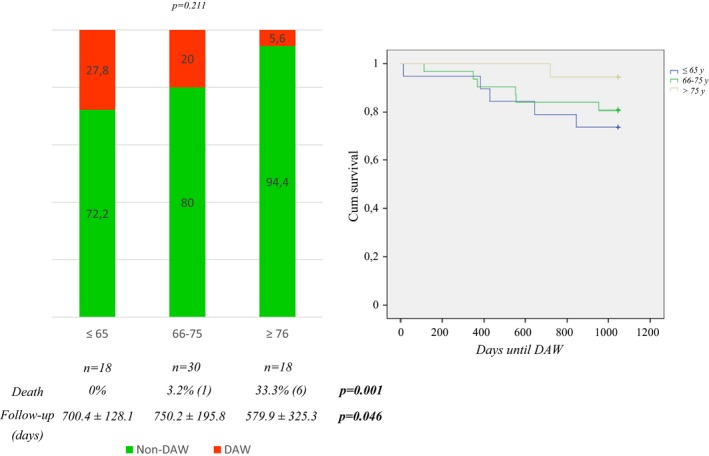
DAW, deaths and follow‐up time in the subgroups of PD patients in relation to age: 1) ≤65 years (5 DAW cases); 2) 66–75 years (6 DAW cases and 1 death); 3) >75 years (DAW in only 1 case and 6 deaths). DAW, dopamine agonist withdrawal.

As shown in Table 2, an earlier age at PD onset, a longer disease duration, a higher daily dose of levodopa, a greater number of pills for PD, polypharmacy, and a higher number of nonmotor symptoms were associated with DAW. However, age was not related to DAW. In fact, only one patient discontinued DA therapy in the subgroup of patients over the age of 75 years old (Fig. 3). On the contrary, mortality was significantly higher in this group of patients.

**Table 2:** Factors related (at baseline) to DAW (*n* = 12) versus non‐DAW (*n* = 54)

	Non‐DAW (*n* = 54)	DAW (*n* = 12)	*P*
Age	70.3 ± 9.1	65.3 ± 8.8	0.092
Males (%)	63	58.3	0.505
Age of symptoms onset	63.5 ± 7.9	54.8 ± 13.1	**0.004**
Disease durarion (years)	7.3 ± 3.9	10.6 ± 8.4	**0.040**
Hoehn&Yahr (ON)	2 [2, 2.5]	2 [1.6, 2]	0.171
UPDRS‐III (ON)	15.3 ± 7.6	15.4 ± 9.5	0.974
UPDRS‐IV	3.1 ± 2.7	4.3 ± 2.7	0.176
Motor fluctuations (%)	38.9	50	0.347
Dyskinesia (%)	40.7	58.3	0.215
Levodopa (%)	85.2	100	0.181
COMT‐inhibitor (%)	53.7	25	0.068
MAO B inhibitor (%)	33.3	25	0.425
Time taking DA therapy (days)	1288.3 ± 805.7	1078.4 ± 812.6	0.419
Levodopa daily dose (mg)	490.7 ± 321.1	820.8 ± 520.7	**0.006**
DA equivalent daily dose (mg)	246.6 ± 117.1	253.8 ± 179.2	0.864
Levodopa equivalent daily dose (mg)	795.2 ± 432	1089.2 ± 641.7	0.064
Comorbidity	4.1 ± 2.6	4 ± 2.6	0.896
Total number of pills for PD	5.6 ± 2.6	7.7 ± 3.6	**0.019**
Polypharmacy	9.4 ± 3.4	12 ± 4.4	**0.028**
NMSS total score (0–360)	65.5 ± 54.1	90.8 ± 58.3	0.154
NMSS domains 2+3+4	26.2 ± 25.6	34.9 ± 26	0.293
Number of nonmotor symptoms	14.9 ± 5.7	19.3 ± 6.3	**0.022**
BDI (0–63)	11.9 ± 7.6	15.1 ± 9	0.214
ADLS (0–100)	80 [60, 90]	65 [35, 80]	0.112

ADLS, Activities of Daily Living Score; BDI, Beck Depression Inventory; COMT, catechol‐O‐methyl transferase; DA, dopamine agonist; DAW, dopamine agonist withdrawal; MAO, monoamine oxidase; NMSS, Nonmotor Symptoms Scale; UPDRS, Unified Parkinson's Disease Rating Scale (part III, motor examination; part IV, motor complications).

Bold values: Results are expressed as mean ± SD or %.

Cox‐regression analysis showed that only the levodopa daily dose was a predictor of DAW. Table 3 shows different models in which DAW was the dependent variable and levodopa daily dose, comorbidity, polypharmacy, socio‐demographic patient‐related variables (age and gender), disease‐related variables (disease duration, age of symptoms onset, ON‐H&Y, ON‐UPDRS‐III, UPDRS‐IV, NMSS total score), and confounder variables (time taking DA therapy and follow‐up time) were independent variables. The nonadjusted hazard ratio (HR) of the levodopa daily dose for DAW was 1.002 (95% confidence interval [CI], 1.001–1.003; *P* = 0.006). The levodopa daily dose HR was 1.003 (95% CI, 1.001–1.006; *P* = 0.044) after adjustment for covariates. As expected, when PD patients were classified according to the levodopa daily dose, a significantly higher percentage of DAW was observed in those receiving more than 750 mg per day (42.8% vs. 11.5%; *P* = 0.014) (Table 4).

**Table 3:** Analysis of the factors predicting dopamine agonist withdrawal

Parameter	Univariate (HR; 95% CI; *P*‐value)	Multivariate Cox regression^3^ (HR; 95% CI; *P*‐value)	Multivariate Cox regression^4^ (HR; 95% CI; *P*‐value)	Multivariate Cox regression^5^ (HR; 95% CI; *P*‐value)
Levodopa daily dose^1^	1.002; 1.001–1.003; **0.006**	1.002; 1.001–1.003; **0.001**	1.002; 1.001–1.004; **0.019**	1.003; 1.001–1.006; **0.044**
Age^1^		0.935; 0.883–0.991; **0.023**	1.331; 0.541–3.278; 0.534	2.211; 0.561–8.707; 0.257
Gender		0.830; 0.261–2.644; 0.275	0.831; 0.247–2.797; 0.765	0.241; 0.038–1.516; 0.129
Age at symptoms onset^1^			0.703; 0.282–1.755; 0.450	0.412; 0.102–1.667; 0.214
Disease duration^1^			0.696; 0.274–1.765; 0.445	0.411; 0.102–1.654; 0.211
Time taking DA therapy^1^			1.000; 0.999–1.001; 0.631	0.999; 0.998–1.001; 0.364
Follow‐up time^1^			1.000; 0.997–1.003; 0.927	1.000; 0.995–1.004; 0.846
Comorbidity^1^				1.040; 0.680–1.592; 0.856
Polypharmacy^1^				1.243; 0.929–1.663; 0.143

^1^Hazard ratio is the risk multiplier per unit increase in the parameter.

^2^Figures in bold are statistically significant (*P* < 0.005).

^3^Variables entered: levodopa daily dose, age and gender.

^4^Variables entered: levodopa daily dose, age, gender, age at diagnosis, disease duration, time taking DA therapy and follow‐up time.

^5^Variables entered: levodopa daily dose, age, gender, age at diagnosis, disease duration, time taking DA therapy, follow‐up time, comorbidity, polypharmacy, and other covariates (ON‐H&Y, ON‐UPRDS‐III, UPDRS‐IV, NMSS total score).

**Table 4:** Percentage of patients who presented dopamine agonist withdrawal (DAW) according to levodopa daily dose (>750 mg vs. ≤750 mg): 42.9% (6/14) vs. 11.5% (6/52); *P* = 0.014

	Levodopa dose ≤ 750 mg/day	Levodopa dose > 750 mg/day	Number of patients
Non DAW	46 (88.5%)	8 (57.1%)	54
DAW	6 (**11.5%**)	6 (**42.9%**)	12
	52	14	66

Bold values: Results are expressed as number of patients and %.

## Discussion

There is considerable controversy regarding the use of DAs in the elderly because of the concern for a higher rate of side effects. However, there is no evidence for such concerns because few individuals older than 75 years have been enrolled in clinical trials for antiparkinsonian medications (Mitchell et al. 1997; Fitzsimmons et al. 2012). Our study demonstrates that an advanced age alone is not a contraindication for the administration of DA therapy. In PD, therapeutic decisions should be individualized, and we must keep in mind that the risk of DAW is higher when DA therapy is administered to PD patients with a longer disease duration, polypharmacy, and a higher daily dose of levodopa.

Clinical–pathological studies have demonstrated that cognitive disability, visual hallucinations, or residential care is more frequent in PD patients over the age of 70 years (Kempster et al. 2007). In addition, older patients metabolize drugs differently than younger patients and may be more susceptible to drug side effects, which are often complicated by comorbid conditions (Robertson et al. 1989). For these reasons, DAs historically have been avoided by some clinicians as therapy in elderly patients. However, Shulman et al. (2000) reported that DAs were well tolerated by very elderly (≥80 years old) and appropriately selected PD patients in clinical practice. In our study, about half of the PD patients were early elderly (from 65 to 74 years old) and a quarter were late elderly (over 75 years old). Furthermore, Oertel et al. (2013) observed that rotigotine was generally well tolerated regardless of age. Our results are in line with these studies.

Treatment considerations for elderly PD patients typically have centered on chronological age, and somewhat arbitrary cutoffs have been established for determining whether DA therapy should be used. However, patients with similar chronological ages may differ substantially with respect to functional or physiological age. Functional age can be assessed by measuring motor and nonmotor symptoms, including cognition, mood, behavior, disability, comorbidity, and other dimensions that contribute to functionality. When considering functional age in treatment planning, the clinician can appreciate variability among patients of the same age (Silver 2006). Our results support this idea. The risk of DAW seems to be higher in PD patients with longer disease duration, polypharmacy and more nonmotor symptoms. Indeed, levodopa dosage determines the probability of DAW, with a thirty percent higher risk for every increase in 1.000 mg of levodopa. In other words, almost half of the patients receiving more than 750 mg per day of levodopa discontinued with DA therapy versus one in ten of patients receiving a smaller dose. Related to this finding, compliance with once‐daily DA therapy has been reported to be suboptimal and to depend upon the total daily dose of levodopa and the total number of daily drugs (Santos‐García et al. 2012). Although comorbidity is a risk factor for the development of somnolence, edema, or hallucinations in PD patients (Biglan et al. 2007), these conditions were not related to DAW in our study. No statistical determinants for the discontinuation of DAs were detected in other studies (Arbouw et al. 2008).

Overall, our findings suggest that DA therapy is well tolerated in properly selected PD patients in clinical practice. The number of patients who discontinued DA therapy in our series was low. In fact, DAW in three cases was not due to adverse events. Moreover, adverse events in three other cases were in the context of cognitive impairment progressing to dementia. Previous observational studies have largely focused on discontinuation rates for DAs introduced as a supplement to levodopa (Hauser et al. 2007; Arbouw et al. 2008, 2009; Parkinson Study Group 2009; Valldeoriola et al. 2009; Nissen et al. 2012). A maintenance of pramipexole in approximately 80% at 6 years (Parkinson Study Group 2009) and 90% between 5 and 10 years for ropinirole (Hauser et al. 2007) has been reported in extended open‐label observational studies. Future studies are needed to know the extent to which PD patients tolerate different DAs according to age (Oertel et al. 2013), and interestingly, the AD type (Kim et al. 2015) and genetic determinants (Arbouw et al. 2007).

This study has several limitations. First, the sample size was small, and as result, some differences between groups might have been insignificant. Second, death is a confounding factor because there were more cases in older patients, and follow‐up time was significantly shorter in older patients. However, the follow‐up time was included as a covariate in the model. Third, we used a surrogate marker of comorbidity, but we think that, in any case, it provides proper information on the variable (a larger number of drugs different of antiparkinsonian agents indicates more frequent and/or severe comorbidity). Fourth, some information may have been missed due to the methodology used (chart review). However, the findings are novel and have practical applicability in clinical practice. Fifth, a cognition assessment using a specific scale at baseline was not conducted. Finally, follow‐up information on adverse events was not collected in patients who continued receiving DA therapy at the end of the follow‐up. Nevertheless, this study was not designed to evaluate tolerability and effectiveness. Thus, we can assume that patients continued taking the medication in clinical practice because it is useful and well tolerated.

In conclusion, our study shows that DA therapy is generally well tolerated by nondemented PD patients in clinical practice. Advanced age alone is not a contraindication to the administration of DA therapy. We must keep in mind that the probability of successful treatment when we use DA therapy is lower in PD patients with a longer disease duration, polypharmacy, and a higher daily dose of levodopa.

## Conflict of Interest

None declared.

## References

[brb3453-bib-0001] Antonini, A. , E. Tolosa , Y. Mizuno , M. Yamamoto , and W. H. Poewe . 2009 A reassessment of risk and benefits of dopamine agonists in Parkinson′s disease. Lancet Neurol. 8:929–937. 19709931 10.1016/S1474-4422(09)70225-X

[brb3453-bib-0002] Arbouw, M. E. , J. P. van Vugt , T. C. Egberts , and H. J. Guchelaar . 2007 Pharmacogenetics of antiparkinsonian drug treatment: a systematic review. Pharmacogenomics 8:159–176. 17286539 10.2217/14622416.8.2.159

[brb3453-bib-0003] Arbouw, M. E. , K. L. Movig , H. J. Guchejaar , P. J. Poels , J. P. van Vugt , C. Neef , et al. 2008 Discontinuation of ropinirole and pramipexole in patients with Parkinson's disease in clinical practice versus clinical trials. Eur. J. Clin. Pharmacol. 64:1021–1026. 18626634 10.1007/s00228-008-0518-2

[brb3453-bib-0004] Arbouw, M. E. , K. L. Movig , T. C. Egberts , P. J. Poels , J. P. van Vugt , J. A. Wessels , et al. 2009 Clinical and pharmacogenetic determinants for the discontinuation of non‐ergoline dopamine agonists in Parkinson's disease. Eur. J. Clin. Pharmacol. 65:1245–1251. 19669131 10.1007/s00228-009-0708-6 PMC2778789

[brb3453-bib-0005] Biglan, K. M. , R. G. Holloway Jr , M. P. McDermott , I. H. Richard , and Parkinson Study Group CALM‐PD Investigators . 2007 Risk factors for somnolence, edema, and hallucinations in early Parkinson disease. Neurology 69:187–195. 17620552 10.1212/01.wnl.0000265593.34438.00

[brb3453-bib-0006] Fitzsimmons, P. R. , S. Blayney , S. Mina‐Corkill , and G. O. Scott . 2012 Older participants are frequently excluded from Parkinson's disease research. Parkinsonism Relat. Disord. 18:585–589. 22494661 10.1016/j.parkreldis.2012.03.003

[brb3453-bib-0007] Hauser, R. A. , O. Rascol , A. D. Korczyn , A. Jon Soessl , R. L. Watts , W. Poewe , et al. 2007 Ten‐year follow‐up of Parkinson's disease patients randomized to initial therapy with ropinirole or levodopa. Mov. Disord. 22:2409–2417. 17894339 10.1002/mds.21743

[brb3453-bib-0008] Kempster, P. A. , D. R. Williams , M. Selikhova , J. Holton , T. Revesz , and A. J. Lees . 2007 Patterns of levodopa response in Parkinson's disease: a clinico‐pathological study. Brain 130:2123–2128. 17586867 10.1093/brain/awm142

[brb3453-bib-0009] Kim, J. M. , S. J. Chung , J. W. Kim , B. S. Jeon , P. Singh , S. Thierfelder , et al. 2015 Rotigotine transdermal system as add‐on to oral dopamine agonist in advanced Parkinson's disease: an open‐label study. BMC Neurol. doi: 10.1186/s12883‐015‐0267‐7 10.1186/s12883-015-0267-7 PMC4364324 25879416

[brb3453-bib-0010] Mitchell, S. L. , E. A. Sullivan , and L. A. Lipsitz . 1997 Exclusion of elderly subjects from clinical trials for Parkinson disease. Arch. Neurol. 54:1393–1398. 9362988 10.1001/archneur.1997.00550230060018

[brb3453-bib-0011] Nissen, T. , E. J. Newman , K. A. Grosset , M. Daghem , G. Pal , M. Stewart , et al. 2012 Duration of L‐dopa and dopamine agonist monotherapy in Parkinson′s disease. Scott. Med. J. 57:217–220. 23002158 10.1258/smj.2012.012121

[brb3453-bib-0012] Oertel, W. , P. LeWitt , N. Giladi , L. Ghys , F. Grieger , and B. Boroojerdi . 2013 Treatment of patients with early and advanced Parkinson's disease with rotigotine transdermal system: age‐relationship to safety and tolerability. Parkinsonism Relat. Disord. 19:37–42. 22954721 10.1016/j.parkreldis.2012.06.009

[brb3453-bib-0013] Parkinson Study Group, CALM Cohort Investigators . 2009 Long‐term effect of initiating pramipexole vs levodopa in early Parkinson disease. Arch. Neurol. 66:563–570. 19433655 10.1001/archneur.66.1.nct90001

[brb3453-bib-0014] Robertson, D. R. , N. D. Wood , H. Everest , K. Monks , D. G. Waller , A. G. Renwick , et al. 1989 The effect of age on the pharmacokinetics of levodopa administered alone and in the presence of carbidopa. Br. J. Clin. Pharmacol. 28:61–69. 2775615 10.1111/j.1365-2125.1989.tb03506.x PMC1379971

[brb3453-bib-0015] Santos‐García, D. , and R. de la Fuente‐Fernández . 2013 Impact of non‐motor symptoms on health‐related and perceived quality of life in Parkinson's disease. J. Neurol. Sci. 332:136–140. 23890935 10.1016/j.jns.2013.07.005

[brb3453-bib-0016] Santos‐García, D. , and R. de la Fuente‐Fernández . 2015 Factors contributing to caregivers' stress and burden in Parkinson's disease. Acta Neurol. Scand. 131:203–210. 25212106 10.1111/ane.12305

[brb3453-bib-0017] Santos‐García, D. , M. Prieto‐Formoso , and R. de la Fuente‐Fernández . 2012 Levodopa dosage determines adherence to long‐acting dopamine agonists in Parkinson's disease. J. Neurol. Sci. 318:90–93. 22521273 10.1016/j.jns.2012.03.018

[brb3453-bib-0018] Shulman, L. M. , A. Minagar , A. Rabinstein , and W. J. Weiner . 2000 The use of dopamine agonists in very elderly patients with Parkinson's disease. Mov. Disord. 15:664–668. 10928576 10.1002/1531-8257(200007)15:4<664::aid-mds1010>3.0.co;2-d

[brb3453-bib-0019] Silver, D. 2006 Impact of functional age on the use of dopamine agonists in patients with Parkinson disease. Neurologist 12:214–223. 16832240 10.1097/01.nrl.0000215782.78763.fa

[brb3453-bib-0020] Stowe, R. L. , N. J. Ives , C. Clarke , J. van Hilten , J. Ferreira , R. J. Hawker , et al. 2008 Dopamine agonist therapy in early Parkinson′s disease. Cochrane Database Syst. Rev.:CD006564. 18425954 10.1002/14651858.CD006564.pub2 PMC12740267

[brb3453-bib-0021] Tomlinson, C. L. , R. Stowe , S. Patel , C. Rick , R. Gray , and C. E. Clarke . 2010 Systematic review of levodopa dose equivalency reporting in Parkinson's disease. Mov. Disord. 25:2649–2653. 21069833 10.1002/mds.23429

[brb3453-bib-0022] Valldeoriola, F. , S. Cobaledac , and J. Lahuertac . 2009 A multicentre retrospective study of the clinical use of ropinirole in the treatment of Parkinson's disease: the ROPI‐PARK study. Clin. Neurol. Neurosurg. 111:742–747. 19733003 10.1016/j.clineuro.2009.07.018

